# Supplementary material for: Activation of the myosin motors in fast‐twitch muscle of the mouse is controlled by mechano‐sensing in the myosin filaments
Source: J Physiol. 2022 Aug 14;600(17):3983–4000. doi: 10.1113/JP283048 (PMC9544795; doi:10.1113/JP283048)
Supplement: Supplementary file 2 — Statistical Summary Document [file TJP-600-3983-s002.docx]

**Manuscript Title:** Activation of the myosin motors in fast-twitch muscle of the mouse is controlled by mechano-sensing in the myosin filaments

**Authors:** Cameron Hill, Elisabetta Brunello, Luca Fusi, Jesús Garcia Ovejero, Malcolm Irving

**Animal model used, if applicable:** Male C57BL/6J mice

**Underlying hypothesis:** This investigation tests the hypothesis that low-load shortening at the start of electrical stimulation delays activation of the thick filament of mouse fast-twitch skeletal muscle when measured with time-resolved X-ray diffraction and compared against fixed-end conditions

Question 1: How does the half-time of a given structural signal differ to that of force generation in fixed-end and low-load shortening protocols?

Question 2: Do mechanical and structural signals differ between fixed-end and low-load shortening protocols at a given time point?

Question 3: When do mechanical and structural signals differ to resting values at given time-points?

**Statistical summary table:**

| **Experimental question number** | **Finding/ conclusion** | **Experimental Variable** | **Mean value** | **SD** | **n** | **P** | **Units** | **Data comparisons** | **Statistical test** | **Any other variable** | **Figure/ table** | **Comments** |
| --- | --- | --- | --- | --- | --- | --- | --- | --- | --- | --- | --- | --- |
| 1. Half-time of X-ray with respect to force increase | Low-load shortening delayed by ~5ms, same time course as fixed-end | Fixed-end EDL *S*_M6_ t_1/2_ | 8.1 | 1.5 | 5 | **0.0431** | ms | *S*_M6_ vs Fixed-end Force | Wilcoxon signed-ranked | Compared against fixed-end force t_1/2_ 17.0 ± 1.5 ms | Table 1 | Shapiro-Wilk P=0.0037 |
|  |  | Fixed-end EDL *A*_ML1_ t_1/2_ | 8.6 | 2.1 | 5 | **0.0003** | ms | *A*_ML1_ vs Fixed-end Force | Student’s t-test, paired |  |  | N/A |
|  |  | Fixed-end EDL *M*_M3_ t_1/2_ | 10.4 | 1.2 | 5 | **<0.0001** | ms | *M*_M3_ vs Fixed-end Force | Student’s t-test, paired |  |  | N/A |
|  |  | Fixed-end EDL *L*_M3_ t_1/2_ | 10.8 | 1.5 | 5 | **0.0003** | ms | *L*_M3_ vs Fixed-end Force | Student’s t-test, paired |  |  | N/A |
|  |  | Fixed-end EDL *I*_1,1_/*I*_1,0_ t_1/2_ | 11.3 | 1.6 | 5 | **0.0001** | ms | *I*_1,1_/*I*_1,0_ vs Fixed-end Force | Student’s t-test, paired |  |  | N/A |
|  |  | Fixed-end EDL *hbz* t_1/2_ | 11.6 | 0.1 | 5 | **0.0017** | ms | *hbz* vs Fixed-end Force | Student’s t-test, paired |  |  | N/A |
|  |  | Fixed-end EDL *S*_M3_ t_1/2_ | 12.4 | 1.8 | 5 | **0.0001** | ms | *S*_M3_ vs Fixed-end Force | Student’s t-test, paired |  |  | N/A |
|  |  | Fixed-end EDL *A*_AL1_ t_1/2_ | 15.3 | 2.6 | 5 | 0.686 | ms | *A*_AL1_ vs Fixed-end Force | Wilcoxon signed-ranked |  |  | Shapiro-Wilk P=0.0024 |
|  |  | Fixed-end EDL *A*_M3_ t_1/2_ | 20.8 | 2.5 | 5 | **0.0431** | ms | *A*_M3_ vs Fixed-end Force | Wilcoxon signed-ranked |  |  | Shapiro-Wilk P=0.0418 |
|  |  | Low-load EDL *S*_M6_ t_1/2_ | 14.5 | 0.6 | 5 | **<0.0001** | ms | *S*_M6_ vs Low-load Force | Student’s t-test, paired | Compared against low-load force t_1/2_ 22.4 ± 0.8 ms | Table 1 | N/A |
|  |  | Low-load EDL *A*_ML1_ t_1/2_ | 15.1 | 1.3 | 5 | **0.0001** | ms | *A*_ML1_ vs Low-load Force | Student’s t-test, paired |  |  | N/A |
|  |  | Low-load EDL *M*_M3_ t_1/2_ | 16.8 | 0.4 | 5 | **<0.0001** | ms | *M*_M3_ vs Low-load Force | Student’s t-test, paired |  |  | N/A |
|  |  | Low-load EDL *L*_M3_ t_1/2_ | 16.8 | 0.3 | 5 | **<0.0001** | ms | *L*_M3_ vs Low-load Force | Student’s t-test, paired |  |  | N/A |
|  |  | Low-load EDL *I*_1,1_/*I*_1,0_ t_1/2_ | 17.5 | 1.3 | 5 | **0.0006** | ms | *I*_1,1_/*I*_1,0_ vs Low-load Force | Student’s t-test, paired |  |  | N/A |
|  |  | Low-load EDL *hbz* t_1/2_ | 16.4 | 0.03 | 5 | **0.0431** | ms | *hbz* vs Fixed-end Force | Wilcoxon signed-ranked |  |  | Shapiro-Wilk P=0.0151 |
|  |  | Low-load EDL *S*_M3_ t_1/2_ | 18.7 | 0.7 | 5 | **0.0016** | ms | *S*_M3_ vs Low-load Force | Student’s t-test, paired |  |  | N/A |
|  |  | Low-load EDL *A*_AL1_ t_1/2_ | 23.3 | 1.2 | 3 | 0.271 | ms | *A*_AL1_ vs Low-load Force | Student’s t-test, paired |  |  | N/A |
|  |  | Low-load EDL *A*_M3_ t_1/2_ | 25.7 | 3.5 | 5 | 0.129 | ms | *A*_M3_ vs Low-load Force | Student’s t-test, paired |  |  | N/A |
| 2. Differences in mechanical and structural signals between protocols at a given time point |  | Rest *T*/*T*_0_ Fixed-end | 0 | 0 | 5 | - | N/A |  |  |  |  |  |
|  |  | Rest *T*/*T*_0_ Low-load | 0 | 0 | 5 |  |  |  |  |  |  |  |
|  |  | 6.5ms *T*/*T*_0_ Fixed-end |  |  | 5 |  |  |  |  |  |  |  |
|  |  | 6.5ms *T*/*T*_0_ Low-load |  |  | 5 |  |  |  |  |  |  |  |
|  |  | 11.5ms *T*/*T*_0_ Fixed-end |  |  | 5 |  |  |  |  |  |  |  |
|  |  | 11.5ms *T*/*T*_0_ Low-load |  |  | 5 |  |  |  |  |  |  |  |
|  |  | 16.5ms *T*/*T*_0_ Fixed-end |  |  | 5 |  |  |  |  |  |  |  |
|  |  | 16.5ms *T*/*T*_0_ Low-load |  |  | 5 |  |  |  |  |  |  |  |
|  |  | *T*_0_ *T*/*T*_0_ Fixed-end | 1 | 0 | 5 | - |  |  |  |  |  |  |
|  |  | *T*_0_ *T*/*T*_0_ Low-load | 1 | 0 | 5 |  |  |  |  |  |  |  |
|  | Low-load *d*_1,0_ lower at rest but no difference thereafter | Rest *d*_1,0_ Fixed-end | 35.32 | 0.24 | 5 | **0.0103** | nm | Fixed-end vs Low-Load for *d*_1,0_ | Student’s t-test, unpaired | N/A | Table 2 | N/A |
|  |  | Rest *d*_1,0_ Low-load | 34.13 | 0.75 | 5 |  |  |  |  |  |  |  |
|  |  | 6.5ms *d*_1,0_ Fixed-end | 35.86 | 0.38 | 5 | 0.181 |  |  |  |  |  |  |
|  |  | 6.5ms *d*_1,0_ Low-load | 35.16 | 0.71 | 5 |  |  |  |  |  |  |  |
|  |  | 11.5ms *d*_1,0_ Fixed-end | 35.84 | 0.38 | 5 | 0.734 |  |  |  |  |  |  |
|  |  | 11.5ms *d*_1,0_ Low-load | 36.10 | 0.65 | 5 |  |  |  |  |  |  |  |
|  |  | 16.5ms *d*_1,0_ Fixed-end | 36.00 | 0.39 | 5 | 0.773 |  |  |  |  |  |  |
|  |  | 16.5ms *d*_1,0_ Low-load | 36.10 | 0.65 | 5 |  |  |  |  |  |  |  |
|  |  | *T*_0_ *d*_1,0_ Fixed-end | 35.75 | 0.36 | 5 | 0.370 |  |  |  |  |  |  |
|  |  | *T*_0_ *d*_1,0_ Low-load | 36.05 | 0.59 | 5 |  |  |  |  |  |  |  |
|  | Low-load significantly higher at 6.5ms but no difference thereafter | 6.5ms *I*_1,0_ Fixed-end | 0.82 | 0.08 | 5 | **0.0067** | N/A | Fixed-end vs Low-Load for *I*_1,0_ | Student’s t-test, unpaired | N/A | Table 2 | N/A |
|  |  | 6.5ms *I*_1,0_ Low-load | 0.96 | 0.03 | 5 |  |  |  |  |  |  |  |
|  |  | 11.5ms *I*_1,0_ Fixed-end | 0.66 | 0.16 | 5 | 0.226 |  |  |  |  |  |  |
|  |  | 11.5ms *I*_1,0_ Low-load | 0.84 | 0.09 | 5 |  |  |  |  |  |  |  |
|  |  | 16.5ms *I*_1,0_ Fixed-end | 0.56 | 0.18 | 5 | 0.725 |  |  |  |  |  |  |
|  |  | 16.5ms *I*_1,0_ Low-load | 0.62 | 0.14 | 5 |  |  |  |  |  |  |  |
|  |  | *T*_0_ *I*_1,0_ Fixed-end | 0.48 | 0.14 | 5 | 0.542 |  |  |  |  |  |  |
|  |  | *T*_0_ *I*_1,0_ Low-load | 0.36 | 0.16 | 5 |  |  |  |  |  |  |  |
|  |  | Rest *I*_1,1_/*I*_1,0_ Fixed-end | 0.41 | 0.04 | 5 | **0.033** | N/A | Fixed-end vs Low-Load for *I*_1,1_/*I*_1,0_ | Student’s t-test, unpaired | N/A | Table 2 | N/A |
|  |  | Rest *I*_1,1_/*I*_1,0_ Low-load | 0.29 | 0.09 | 5 |  |  |  |  |  |  |  |
|  |  | 6.5ms *I*_1,1_/*I*_1,0_ Fixed-end | 0.77 | 0.09 | 5 | **0.0008** |  |  | Student’s t-test, unpaired |  |  | N/A |
|  |  | 6.5ms *I*_1,1_/*I*_1,0_ Low-load | 0.44 | 0.11 | 5 |  |  |  |  |  |  |  |
|  |  | 11.5ms *I*_1,1_/*I*_1,0_ Fixed-end | 1.19 | 0.27 | 5 | **0.0032** |  |  | Student’s t-test, unpaired |  |  | N/A |
|  |  | 11.5ms *I*_1,1_/*I*_1,0_ Low-load | 0.66 | 0.11 | 5 |  |  |  |  |  |  |  |
|  |  | 16.5ms *I*_1,1_/*I*_1,0_ Fixed-end | 1.45 | 0.32 | 5 | **0.0280** |  |  | Mann-Whitney U test |  |  | Levene’s P=0.0427 |
|  |  | 16.5ms *I*_1,1_/*I*_1,0_ Low-load | 0.98 | 0.13 | 5 |  |  |  |  |  |  |  |
|  |  | *T*_0_ *I*_1,1_/*I*_1,0_ Fixed-end | 1.77 | 0.31 | 5 | 0.488 |  |  | Student’s t-test, unpaired |  |  | N/A |
|  |  | *T*_0_ *I*_1,1_/*I*_1,0_ Low-load | 1.92 | 0.34 | 5 |  |  |  |  |  |  |  |
|  |  | 6.5ms *I*_ML1_ Fixed-end | 0.61 | 0.23 | 5 | **0.0355** | N/A | Fixed-end vs Low-Load for *I*_ML1_ | Student’s t-test, unpaired | N/A | Table 2 | N/A |
|  |  | 6.5ms *I*_ML1_ Low-load | 0.91 | 0.12 | 5 |  |  |  |  |  |  |  |
|  |  | 11.5ms *I*_ML1_ Fixed-end | 0.23 | 0.13 | 5 | **0.0004** |  |  |  |  |  |  |
|  |  | 11.5ms *I*_ML1_ Low-load | 0.66 | 0.11 | 5 |  |  |  |  |  |  |  |
|  |  | 16.5ms *I*_ML1_ Fixed-end | 0.12 | 0.15 | 5 | 0.0907 |  |  |  |  |  |  |
|  |  | 16.5ms *I*_ML1_ Low-load | 0.27 | 0.10 | 5 |  |  |  |  |  |  |  |
|  |  | *T*_0_ *I*_ML1_ Fixed-end | 0.09 | 0.05 | 5 | 0.0831 |  |  |  |  |  |  |
|  |  | *T*_0_ *I*_ML1_ Low-load | 0.10 | 0.08 | 5 |  |  |  |  |  |  |  |
|  |  | 6.5ms *A*_ML1_ Fixed-end | 0.77 | 0.15 | 5 | **0.0355** | N/A | Fixed-end vs Low-Load for *I*_ML1_ | Student’s t-test, unpaired | N/A | Table 2 | N/A |
|  |  | 6.5ms *A*_ML1_ Low-load | 0.95 | 0.07 | 5 |  |  |  |  |  |  |  |
|  |  | 11.5ms *A*_ML1_ Fixed-end | 0.47 | 0.12 | 5 | **0.0004** |  |  |  |  |  |  |
|  |  | 11.5ms *A*_ML1_ Low-load | 0.81 | 0.07 | 5 |  |  |  |  |  |  |  |
|  |  | 16.5ms *A*_ML1_ Fixed-end | 0.38 | 0.15 | 5 | 0.0907 |  |  |  |  |  |  |
|  |  | 16.5ms *A*_ML1_ Low-load | 0.51 | 0.10 | 5 |  |  |  |  |  |  |  |
|  |  | *T*_0_ *A*_ML1_ Fixed-end | 0.34 | 0.11 | 5 | 0.0831 |  |  |  |  |  |  |
|  |  | *T*_0_ *A*_ML1_ Low-load | 0.20 | 0.07 | 5 |  |  |  |  |  |  |  |
|  |  | 6.5ms *I*_AL1_ Fixed-end | 1.02 | 0.46 | 5 | 0.636 | N/A | Fixed-end vs Low-Load for *I*_AL1_ | Student’s t-test, unpaired | N/A | Table 2 | N/A |
|  |  | 6.5ms *I*_AL1_ Low-load | 0.85 | 0.66 | 5 |  |  |  |  |  |  |  |
|  |  | 11.5ms *I*_AL1_ Fixed-end | 1.44 | 0.94 | 5 | 0.360 |  |  |  |  |  |  |
|  |  | 11.5ms *I*_AL1_ Low-load | 0.99 | 0.46 | 5 |  |  |  |  |  |  |  |
|  |  | 16.5ms *I*_AL1_ Fixed-end | 1.90 | 0.87 | 5 | 0.121 |  |  |  |  |  |  |
|  |  | 16.5ms *I*_AL1_ Low-load | 1.05 | 0.68 | 5 |  |  |  |  |  |  |  |
|  |  | *T*_0_ *I*_AL1_ Fixed-end | 2.68 | 1.11 | 5 | 0.914 |  |  |  |  |  |  |
|  |  | *T*_0_ *I*_AL1_ Low-load | 2.58 | 1.64 | 5 |  |  |  |  |  |  |  |
|  |  | 6.5ms *A*_AL1_ Fixed-end | 0.99 | 0.24 | 5 | 0.525 | N/A | Fixed-end vs Low-Load for *A*_AL1_ | Student’s t-test, unpaired | N/A | Table 2 | N/A |
|  |  | 6.5ms *A*_AL1_ Low-load | 0.86 | 0.37 | 5 |  |  |  |  |  |  |  |
|  |  | 11.5ms *A*_AL1_ Fixed-end | 1.16 | 0.35 | 5 | 0.340 |  |  |  |  |  |  |
|  |  | 11.5ms *A*_AL1_ Low-load | 0.97 | 0.23 | 5 |  |  |  |  |  |  |  |
|  |  | 16.5ms *A*_AL1_ Fixed-end | 1.36 | 0.29 | 5 | 0.098 |  |  |  |  |  |  |
|  |  | 16.5ms *A*_AL1_ Low-load | 0.98 | 0.35 | 5 |  |  |  |  |  |  |  |
|  |  | *T*_0_ *A*_AL1_ Fixed-end | 1.61 | 0.33 | 5 | 0.650 |  |  |  |  |  |  |
|  |  | *T*_0_ *A*_AL1_ Low-load | 1.52 | 0.57 | 5 |  |  |  |  |  |  |  |
|  |  | 6.5ms *I*_M6_ Fixed-end | 1.16 | 0.18 | 5 | 0.516 | N/A | Fixed-end vs Low-Load for *I*_M6_ | Student’s t-test, unpaired | N/A | Table 2 | N/A |
|  |  | 6.5ms *I*_M6_ Low-load | 1.05 | 0.33 | 5 |  |  |  |  |  |  |  |
|  |  | 11.5ms *I*_M6_ Fixed-end | 1.01 | 0.22 | 5 | 0.870 |  |  |  |  |  |  |
|  |  | 11.5ms *I*_M6_ Low-load | 1.04 | 0.32 | 5 |  |  |  |  |  |  |  |
|  |  | 16.5ms *I*_M6_ Fixed-end | 1.03 | 0.46 | 5 | 0.956 |  |  |  |  |  |  |
|  |  | 16.5ms *I*_M6_ Low-load | 1.06 | 0.84 | 5 |  |  |  |  |  |  |  |
|  |  | *T*_0_ *I*_M6_ Fixed-end | 1.09 | 0.47 | 5 | 0.644 |  |  |  |  |  |  |
|  |  | *T*_0_ *I*_M6_ Low-load | 1.53 | 0.98 | 5 |  |  |  |  |  |  |  |
|  |  | Rest *S*_M6_ Fixed-end | 7.173 | 0.005 | 5 | 0.305 | nm | Fixed-end vs Low-Load for *S*_M6_ | Student’s t-test, unpaired | N/A | Table 2 | N/A |
|  |  | Rest *S*_M6_ Low-load | 7.176 | 0.003 | 5 |  |  |  |  |  |  |  |
|  |  | 6.5ms *S*_M6_ Fixed-end | 7.222 | 0.012 | 5 | **0.0003** |  |  |  |  |  |  |
|  |  | 6.5ms *S*_M6_ Low-load | 7.188 | 0.004 | 5 |  |  |  |  |  |  |  |
|  |  | 11.5ms *S*_M6_ Fixed-end | 7.253 | 0.009 | 5 | **<0.0001** |  |  |  |  |  |  |
|  |  | 11.5ms *S*_M6_ Low-load | 7.202 | 0.005 | 5 |  |  |  |  |  |  |  |
|  |  | 16.5ms *S*_M6_ Fixed-end | 7.268 | 0.011 | 5 | **0.0070** |  |  |  |  |  |  |
|  |  | 16.5ms *S*_M6_ Low-load | 7.244 | 0.009 | 5 |  |  |  |  |  |  |  |
|  |  | *T*_0_ *S*_M6_ Fixed-end | 7.283 | 0.003 | 5 | 0.307 |  |  |  |  |  |  |
|  |  | *T*_0_ *S*_M6_ Low-load | 7.280 | 0.006 | 5 |  |  |  |  |  |  |  |
|  |  | 6.5ms *I*_M3_ Fixed-end | 0.75 | 0.16 | 5 | 0.588 |  | Fixed-end vs Low-Load for *I*_M3_ | Student’s t-test, unpaired | N/A | Table 2 | N/A |
|  |  | 6.5ms *I*_M3_ Low-load | 0.80 | 0.12 | 5 |  |  |  |  |  |  |  |
|  |  | 11.5ms *I*_M3_ Fixed-end | 0.99 | 0.04 | 5 | **0.0180** |  |  | Mann-Whitney U test |  |  | Levene’s P=0.0009 |
|  |  | 11.5ms *I*_M3_ Low-load | 0.73 | 0.15 | 5 |  |  |  |  |  |  |  |
|  |  | 16.5ms *I*_M3_ Fixed-end | 1.54 | 0.13 | 5 | 0.242 |  |  | Mann-Whitney U test |  |  | Levene’s P=0.0182 |
|  |  | 16.5ms *I*_M3_ Low-load | 1.08 | 0.75 | 5 |  |  |  |  |  |  |  |
|  |  | *T*_0_ *I*_M3_ Fixed-end | 3.18 | 0.56 | 5 | 0.607 |  |  | Mann-Whitney U test |  |  | Levene’s P=0.0017 |
|  |  | *T*_0_ *I*_M3_ Low-load | 3.86 | 2.67 | 5 |  |  |  |  |  |  |  |
|  |  | 6.5ms *A*_M3_ Fixed-end | 0.86 | 0.09 | 5 | 0.559 | N/A | Fixed-end vs Low-Load for *A*_M3_ | Student’s t-test, unpaired | N/A | Table 2 | N/A |
|  |  | 6.5ms *A*_M3_ Low-load | 0.89 | 0.06 | 5 |  |  |  |  |  |  |  |
|  |  | 11.5ms *A*_M3_ Fixed-end | 0.99 | 0.02 | 5 | **0.0238** |  |  | Mann-Whitney U test |  |  | Levene’s P=0.0005 |
|  |  | 11.5ms *A*_M3_ Low-load | 0.85 | 0.09 | 5 |  |  |  |  |  |  |  |
|  |  | 16.5ms *A*_M3_ Fixed-end | 1.24 | 0.05 | 5 | 0.194 |  |  | Mann-Whitney U test |  |  | Levene’s P=0.0177 |
|  |  | 16.5ms *A*_M3_ Low-load | 0.99 | 0.36 | 5 |  |  |  |  |  |  |  |
|  |  | *T*_0_ *A*_M3_ Fixed-end | 1.78 | 0.16 | 5 | 0.804 |  |  | Mann-Whitney U test |  |  | Levene’s P=0.0056 |
|  |  | *T*_0_ *A*_M3_ Low-load | 1.86 | 0.70 | 5 |  |  |  |  |  |  |  |
|  |  | Rest *L*_M3_ Fixed-end | 0.04 | 0.02 | 5 | 0.179 | N/A | Fixed-end vs Low-Load for *L*_M3_ | Student’s t-test, unpaired | N/A | Table 2 | N/A |
|  |  | Rest *L*_M3_ Low-load | 0.06 | 0.02 | 5 |  |  |  |  |  |  |  |
|  |  | 6.5ms *L*_M3_ Fixed-end | 0.07 | 0.03 | 5 | **0.0054** |  |  | Mann-Whitney U test |  |  | Levene’s P=0.0155 |
|  |  | 6.5ms *L*_M3_ Low-load | 0 | 0 | 5 |  |  |  |  |  |  |  |
|  |  | 11.5ms *L*_M3_ Fixed-end | 0.38 | 0.12 | 5 | **0.0002** |  |  | Student’s t-test, unpaired |  |  | N/A |
|  |  | 11.5ms *L*_M3_ Low-load | 0.03 | 0.03 | 5 |  |  |  |  |  |  |  |
|  |  | 16.5ms *L*_M3_ Fixed-end | 0.55 | 0.07 | 5 | **<0.0001** |  |  | Student’s t-test, unpaired |  |  | N/A |
|  |  | 16.5ms *L*_M3_ Low-load | 0.26 | 0.05 | 5 |  |  |  |  |  |  |  |
|  |  | *T*_0_ *L*_M3_ Fixed-end | 0.54 | 0.01 | 5 | 0.129 |  |  | Student’s t-test, unpaired |  |  | N/A |
|  |  | *T*_0_ *L*_M3_ Low-load | 0.56 | 0.02 | 5 |  |  |  |  |  |  |  |
|  |  | Rest *M*_M3_ Fixed-end | 0.83 | 0.02 | 5 | 0.228 | N/A | Fixed-end vs Low-Load for *M*_M3_ | Mann-Whitney U test | N/A | Table 2 | Levene’s P=0.0434 |
|  |  | Rest *M*_M3_ Low-load | 0.81 | 0.04 | 5 |  |  |  |  |  |  |  |
|  |  | 6.5ms *M*_M3_ Fixed-end | 0.79 | 0.01 | 5 | **0.0001** |  |  | Student’s t-test, unpaired |  |  | N/A |
|  |  | 6.5ms *M*_M3_ Low-load | 0.84 | 0.02 | 5 |  |  |  |  |  |  |  |
|  |  | 11.5ms *M*_M3_ Fixed-end | 0.54 | 0.08 | 5 | **<0.0001** |  |  | Student’s t-test, unpaired |  |  | N/A |
|  |  | 11.5ms *M*_M3_ Low-load | 0.82 | 0.02 | 5 |  |  |  |  |  |  |  |
|  |  | 16.5ms *M*_M3_ Fixed-end | 0.39 | 0.06 | 5 | **0.0003** |  |  | Student’s t-test, unpaired |  |  | N/A |
|  |  | 16.5ms *M*_M3_ Low-load | 0.64 | 0.07 | 5 |  |  |  |  |  |  |  |
|  |  | *T*_0_ *M*_M3_ Fixed-end | 0.43 | 0.01 | 5 | 0.130 |  |  | Student’s t-test, unpaired |  |  | N/A |
|  |  | *T*_0_ *M*_M3_ Low-load | 0.41 | 0.02 | 5 |  |  |  |  |  |  |  |
|  |  | Rest *H*_M3_ Fixed-end | 0.13 | 0.01 | 5 | 0.487 | N/A | Fixed-end vs Low-Load for *H*_M3_ | Student’s t-test, unpaired | N/A | Table 2 | N/A |
|  |  | Rest *H*_M3_ Low-load | 0.13 | 0.02 | 5 |  |  |  |  |  |  |  |
|  |  | 6.5ms *H*_M3_ Fixed-end | 0.14 | 0.02 | 5 | 0.203 |  |  | Student’s t-test, unpaired |  |  | N/A |
|  |  | 6.5ms *H*_M3_ Low-load | 0.16 | 0.02 | 5 |  |  |  |  |  |  |  |
|  |  | 11.5ms *H*_M3_ Fixed-end | 0.09 | 0.04 | 5 | **0.0293** |  |  | Mann-Whitney U test |  |  | Levene’s P=0.0152 |
|  |  | 11.5ms *H*_M3_ Low-load | 0.15 | 0.01 | 5 |  |  |  |  |  |  |  |
|  |  | 16.5ms *H*_M3_ Fixed-end | 0.05 | 0.02 | 5 | **0.0443** |  |  | Student’s t-test, unpaired |  |  | N/A |
|  |  | 16.5ms *H*_M3_ Low-load | 0.10 | 0.04 | 5 |  |  |  |  |  |  |  |
|  |  | *T*_0_ *H*_M3_ Fixed-end | 0.03 | 0.003 | 5 | 0.945 |  |  | Student’s t-test, unpaired |  |  | N/A |
|  |  | *T*_0_ *H*_M3_ Low-load | 0.04 | 0.01 | 5 |  |  |  |  |  |  |  |
|  |  | Rest *S*_M3_ Fixed-end | 14.339 | 0.008 | 5 | 0.695 | nm | Fixed-end vs Low-Load for *S*_M3_ | Student’s t-test, unpaired | N/A | Table 2 | N/A |
|  |  | Rest *S*_M3_ Low-load | 14.341 | 0.010 | 5 |  |  |  |  |  |  |  |
|  |  | 6.5ms *S*_M3_ Fixed-end | 14.347 | 0.011 | 5 | **0.0003** |  |  |  |  |  |  |
|  |  | 6.5ms *S*_M3_ Low-load | 14.312 | 0.007 | 5 |  |  |  |  |  |  |  |
|  |  | 11.5ms *S*_M3_ Fixed-end | 14.431 | 0.034 | 5 | **<0.0001** |  |  |  |  |  |  |
|  |  | 11.5ms *S*_M3_ Low-load | 14.316 | 0.010 | 5 |  |  |  |  |  |  |  |
|  |  | 16.5ms *S*_M3_ Fixed-end | 14.490 | 0.021 | 5 | **<0.0001** |  |  |  |  |  |  |
|  |  | 16.5ms *S*_M3_ Low-load | 14.394 | 0.014 | 5 |  |  |  |  |  |  |  |
|  |  | *T*_0_ *S*_M3_ Fixed-end | 14.533 | 0.003 | 5 | 0.994 |  |  |  |  |  |  |
|  |  | *T*_0_ *S*_M3_ Low-load | 14.534 | 0.011 | 5 |  |  |  |  |  |  |  |
|  |  | Rest *d* Fixed-end | 14.343 | 0.009 | 5 | 0.977 | nm | Fixed-end vs Low-Load for *d* | Student’s t-test, unpaired | N/A | Table 2 | N/A |
|  |  | Rest *d* Low-load | 14.343 | 0.013 | 5 |  |  |  |  |  |  |  |
|  |  | 6.5ms *d* Fixed-end | 14.354 | 0.009 | 5 | **<0.0001** |  |  |  |  |  |  |
|  |  | 6.5ms *d* Low-load | 14.317 | 0.003 | 5 |  |  |  |  |  |  |  |
|  |  | 11.5ms *d* Fixed-end | 14.443 | 0.042 | 5 | **0.0002** |  |  |  |  |  |  |
|  |  | 11.5ms *d* Low-load | 14.322 | 0.006 | 5 |  |  |  |  |  |  |  |
|  |  | 16.5ms *d* Fixed-end | 14.515 | 0.018 | 5 | **<0.0001** |  |  |  |  |  |  |
|  |  | 16.5ms *d* Low-load | 14.400 | 0.009 | 5 |  |  |  |  |  |  |  |
|  |  | *T*_0_ *d* Fixed-end | 14.552 | 0.003 | 5 |  |  |  |  |  |  |  |
|  |  | *T*_0_ *d* Low-load | 14.550 | 0.007 | 5 |  |  |  |  |  |  |  |
|  |  | Rest *d*_1,0_ Fixed-end | 35.32 | 0.24 | 5 | - | nm | Rest vs 6.5ms, 11.5ms, 16.5ms and *T*_0_ for Fixed-end *d*_1,0_ | Student’s t-test, paired | N/A | Table 2 | N/A |
|  |  | 6.5ms *d*_1,0_ Fixed-end | 35.86 | 0.38 | 5 | **0.0079** |  |  |  |  |  |  |
|  |  | 11.5ms *d*_1,0_ Fixed-end | 35.84 | 0.38 | 5 | **0.0076** |  |  |  |  |  |  |
|  |  | 16.5ms *d*_1,0_ Fixed-end | 36.00 | 0.39 | 5 | **0.0044** |  |  |  |  |  |  |
|  |  | *T*_0_ *d*_1,0_ Fixed-end | 35.75 | 0.36 | 5 | **0.0208** |  |  |  |  |  |  |
|  |  | Rest *I*_1,0_ Fixed-end | 1 | 0 | 5 | - | N/A | Rest vs 6.5ms, 11.5ms, 16.5ms and *T*_0_ for Fixed-end *I*_1,0_ | Student’s t-test, paired | N/A | Table 2 | N/A |
|  |  | 6.5ms *I*_1,0_ Fixed-end | 0.82 | 0.08 | 5 | **0.0067** |  |  |  |  |  |  |
|  |  | 11.5ms *I*_1,0_ Fixed-end | 0.66 | 0.16 | 5 | **0.0083** |  |  |  |  |  |  |
|  |  | 16.5ms *I*_1,0_ Fixed-end | 0.56 | 0.18 | 5 | **0.0059** |  |  |  |  |  |  |
|  |  | *T*_0_ *I*_1,0_ Fixed-end | 0.48 | 0.14 | 5 | **0.0011** |  |  |  |  |  |  |
|  |  | Rest *I*_1,1_/*I*_1,0_ Fixed-end | 0.41 | 0.04 | 5 | - | N/A | Rest vs 6.5ms, 11.5ms, 16.5ms and *T*_0_ for Fixed-end *I*_1,1_/*I*_1,0_ | Student’s t-test, paired | N/A | Table 2 | N/A |
|  |  | 6.5ms *I*_1,1_/*I*_1,0_ Fixed-end | 0.77 | 0.09 | 5 | **0.0013** |  |  |  |  |  |  |
|  |  | 11.5ms *I*_1,1_/*I*_1,0_ Fixed-end | 1.19 | 0.27 | 5 | **0.0031** |  |  |  |  |  |  |
|  |  | 16.5ms *I*_1,1_/*I*_1,0_ Fixed-end | 1.45 | 0.32 | 5 | **0.0019** |  |  |  |  |  |  |
|  |  | *T*_0_ *I*_1,1_/*I*_1,0_ Fixed-end | 1.77 | 0.31 | 5 | **0.0006** |  |  |  |  |  |  |
|  |  | Rest *I*_ML1_ Fixed-end | 1 | 0 | 5 | - | N/A | Rest vs 6.5ms, 11.5ms, 16.5ms and *T*_0_ for Fixed-end *I*_ML1_ | Student’s t-test, paired | N/A | Table 2 | N/A |
|  |  | 6.5ms *I*_ML1_ Fixed-end | 0.61 | 0.23 | 5 | **0.0202** |  |  |  |  |  |  |
|  |  | 11.5ms *I*_ML1_ Fixed-end | 0.23 | 0.13 | 5 | **0.0002** |  |  |  |  |  |  |
|  |  | 16.5ms *I*_ML1_ Fixed-end | 0.12 | 0.15 | 5 | **0.0002** |  |  |  |  |  |  |
|  |  | *T*_0_ *I*_ML1_ Fixed-end | 0.09 | 0.05 | 5 | **<0.0001** |  |  |  |  |  |  |
|  |  | Rest *A*_ML1_ Fixed-end | 1 | 0 | 5 | - | N/A | Rest vs 6.5ms, 11.5ms, 16.5ms and *T*_0_ for Fixed-end *A*_ML1_ | Student’s t-test, paired | N/A | Table 2 | N/A |
|  |  | 6.5ms *A*_ML1_ Fixed-end | 0.77 | 0.15 | 5 | **0.0244** |  |  |  |  |  |  |
|  |  | 11.5ms *A*_ML1_ Fixed-end | 0.47 | 0.12 | 5 | **0.0006** |  |  |  |  |  |  |
|  |  | 16.5ms *A*_ML1_ Fixed-end | 0.38 | 0.15 | 5 | **0.0037** |  |  |  |  |  |  |
|  |  | *T*_0_ *A*_ML1_ Fixed-end | 0.34 | 0.11 | 5 | **0.0002** |  |  |  |  |  |  |
|  |  | Rest *I*_AL1_ Fixed-end | 1 | 0 | 5 | - | N/A | Rest vs 6.5ms, 11.5ms, 16.5ms and *T*_0_ for Fixed-end *I*_AL1_ | N/A | N/A | Table 2 |  |
|  |  | 6.5ms *I*_AL1_ Fixed-end | 1..02 | 0.46 | 5 | 0.916 |  |  | Student’s t-test, paired |  |  |  |
|  |  | 11.5ms *I*_AL1_ Fixed-end | 1.44 | 0.94 | 5 | 0.500 |  |  | Wilcoxon signed rank test |  |  | Shapiro-Wilk P=0.0157 |
|  |  | 16.5ms *I*_AL1_ Fixed-end | 1.90 | 0.87 | 5 | **0.0431** |  |  | Wilcoxon signed rank test |  |  | Shapiro-Wilk P=0.0234 |
|  |  | *T*_0_ *I*_AL1_ Fixed-end | 2.68 | 1.11 | 5 | **0.0276** |  |  | Student’s t-test, paired |  |  |  |
|  |  | Rest *A*_AL1_ Fixed-end | 1 | 0 | 5 | - | N/A | Rest vs 6.5ms, 11.5ms, 16.5ms and *T*_0_ for Fixed-end *A*_AL1_ |  | N/A | Table 2 |  |
|  |  | 6.5ms *A*_AL1_ Fixed-end | 0.99 | 0.24 | 5 | 0.921 |  |  | Student’s t-test, paired |  |  |  |
|  |  | 11.5ms *A*_AL1_ Fixed-end | 1.16 | 0.35 | 5 | 0.361 |  |  | Student’s t-test, paired |  |  |  |
|  |  | 16.5ms *A*_AL1_ Fixed-end | 1.36 | 0.29 | 5 | **0.0431** |  |  | Wilcoxon signed rank test |  |  | Shapiro-Wilk P=0.0401 |
|  |  | *T*_0_ *A*_AL1_ Fixed-end | 1.61 | 0.33 | 5 | **0.0150** |  |  | Student’s t-test, paired |  |  |  |
|  |  | Rest *I*_M6_ Fixed-end | 1 | 0 | 5 | - | N/A | Rest vs 6.5ms, 11.5ms, 16.5ms and *T*_0_ for Fixed-end *I*_M6_ | Student’s t-test, paired | N/A | Table 2 | N/A |
|  |  | 6.5ms *I*_M6_ Fixed-end | 1.16 | 0.18 | 5 | 0.761 |  |  |  |  |  |  |
|  |  | 11.5ms *I*_M6_ Fixed-end | 1.01 | 0.22 | 5 | 0.785 |  |  |  |  |  |  |
|  |  | 16.5ms *I*_M6_ Fixed-end | 1.03 | 0.46 | 5 | 0.882 |  |  |  |  |  |  |
|  |  | *T*_0_ *I*_M6_ Fixed-end | 1.09 | 0.47 | 5 | 0.582 |  |  |  |  |  |  |
|  |  | Rest *S*_M6_ Fixed-end | 7.173 | 0.005 | 5 | - | nm | Rest vs 6.5ms, 11.5ms, 16.5ms and *T*_0_ for Fixed-end *S*_M6_ | Student’s t-test, paired | N/A | Table 2 | N/A |
|  |  | 6.5ms *S*_M6_ Fixed-end | 7.222 | 0.012 | 5 | **0.0003** |  |  |  |  |  |  |
|  |  | 11.5ms *S*_M6_ Fixed-end | 7.253 | 0.009 | 5 | **<0.0001** |  |  |  |  |  |  |
|  |  | 16.5ms *S*_M6_ Fixed-end | 7.268 | 0.011 | 5 | **<0.0001** |  |  |  |  |  |  |
|  |  | *T*_0_ *S*_M6_ Fixed-end | 7.283 | 0.003 | 5 | **<0.0001** |  |  |  |  |  |  |
|  |  | Rest *I*_M3_ Fixed-end | 1 | 0 | 5 | - | N/A | Rest vs 6.5ms, 11.5ms, 16.5ms and *T*_0_ for Fixed-end *I*_M3_ | Student’s t-test, paired | N/A | Table 2 | N/A |
|  |  | 6.5ms *I*_M3_ Fixed-end | 0.75 | 0.16 | 5 | **0.0277** |  |  |  |  |  |  |
|  |  | 11.5ms *I*_M3_ Fixed-end | 0.99 | 0.04 | 5 | 0.570 |  |  |  |  |  |  |
|  |  | 16.5ms *I*_M3_ Fixed-end | 1.54 | 0.13 | 5 | **0.0008** |  |  |  |  |  |  |
|  |  | *T*_0_ *I*_M3_ Fixed-end | 3.18 | 0.56 | 5 | **<0.0001** |  |  |  |  |  |  |
|  |  | Rest *A*_M3_ Fixed-end | 1 | 0 | 5 | - | N/A | Rest vs 6.5ms, 11.5ms, 16.5ms and *T*_0_ for Fixed-end *A*_M3_ | Student’s t-test, paired | N/A | Table 2 | N/A |
|  |  | 6.5ms *A*_M3_ Fixed-end | 0.86 | 0.09 | 5 | **0.0316** |  |  |  |  |  |  |
|  |  | 11.5ms *A*_M3_ Fixed-end | 0.99 | 0.02 | 5 | 0.568 |  |  |  |  |  |  |
|  |  | 16.5ms *A*_M3_ Fixed-end | 1.24 | 0.05 | 5 | **0.0005** |  |  |  |  |  |  |
|  |  | *T*_0_ *A*_M3_ Fixed-end | 1.78 | 0.16 | 5 | **0.0004** |  |  |  |  |  |  |
|  |  | Rest *L*_M3_ Fixed-end | 0.04 | 0.02 | 5 | - | N/A | Rest vs 6.5ms, 11.5ms, 16.5ms and *T*_0_ for Fixed-end *L*_M3_ | Student’s t-test, paired | N/A | Table 2 | N/A |
|  |  | 6.5ms *L*_M3_ Fixed-end | 0.07 | 0.03 | 5 | 0.079 |  |  |  |  |  |  |
|  |  | 11.5ms *L*_M3_ Fixed-end | 0.38 | 0.12 | 5 | **0.0023** |  |  |  |  |  |  |
|  |  | 16.5ms *L*_M3_ Fixed-end | 0.55 | 0.07 | 5 | **<0.0001** |  |  |  |  |  |  |
|  |  | *T*_0_ *L*_M3_ Fixed-end | 0.54 | 0.01 | 5 | **<0.0001** |  |  |  |  |  |  |
|  |  | Rest *M*_M3_ Fixed-end | 0.83 | 0.02 | 5 | - | N/A | Rest vs 6.5ms, 11.5ms, 16.5ms and *T*_0_ for Fixed-end *M*_M3_ | Student’s t-test, paired | N/A | Table 2 | N/A |
|  |  | 6.5ms *M*_M3_ Fixed-end | 0.79 | 0.01 | 5 | **0.0060** |  |  |  |  |  |  |
|  |  | 11.5ms *M*_M3_ Fixed-end | 0.54 | 0.08 | 5 | **0.0010** |  |  |  |  |  |  |
|  |  | 16.5ms *M*_M3_ Fixed-end | 0.39 | 0.06 | 5 | **<0.0001** |  |  |  |  |  |  |
|  |  | *T*_0_ *M*_M3_ Fixed-end | 0.43 | 0.01 | 5 | **<0.0001** |  |  |  |  |  |  |
|  |  | Rest *H*_M3_ Fixed-end | 0.13 | 0.01 | 5 | - | N/A | Rest vs 6.5ms, 11.5ms, 16.5ms and *T*_0_ for Fixed-end *H*_M3_ | Student’s t-test, paired | N/A | Table 2 | N/A |
|  |  | 6.5ms *H*_M3_ Fixed-end | 0.14 | 0.02 | 5 | 0.0793 |  |  |  |  |  |  |
|  |  | 11.5ms *H*_M3_ Fixed-end | 0.09 | 0.04 | 5 | 0.0795 |  |  |  |  |  |  |
|  |  | 16.5ms *H*_M3_ Fixed-end | 0.05 | 0.02 | 5 | **0.0004** |  |  |  |  |  |  |
|  |  | *T*_0_ *H*_M3_ Fixed-end | 0.04 | 0.003 | 5 | **<0.0001** |  |  |  |  |  |  |
|  |  | Rest *S*_M3_ Fixed-end | 14.339 | 0.008 | 5 | - | nm | Rest vs 6.5ms, 11.5ms, 16.5ms and *T*_0_ for Fixed-end *S*_M3_ | Student’s t-test, paired | N/A | Table 2 | N/A |
|  |  | 6.5ms *S*_M3_ Fixed-end | 14.347 | 0.011 | 5 | 0.0575 |  |  |  |  |  |  |
|  |  | 11.5ms *S*_M3_ Fixed-end | 14.431 | 0.021 | 5 | **0.0020** |  |  |  |  |  |  |
|  |  | 16.5ms *S*_M3_ Fixed-end | 14.490 | 0.021 | 5 | **<0.0001** |  |  |  |  |  |  |
|  |  | *T*_0_ *S*_M3_ Fixed-end | 14.533 | 0.003 | 5 | **<0.0001** |  |  |  |  |  |  |
|  |  | Rest *d* Fixed-end | 14.343 | 0.009 | 5 | - | nm | Rest vs 6.5ms, 11.5ms, 16.5ms and *T*_0_ for Fixed-end *d* | Student’s t-test, paired | N/A | Table 2 | N/A |
|  |  | 6.5ms *S*_M3_ Fixed-end | 14.354 | 0.009 | 5 | 0.0677 |  |  |  |  |  |  |
|  |  | 11.5ms *d* Fixed-end | 14.443 | 0.042 | 5 | **0.0042** |  |  |  |  |  |  |
|  |  | 16.5ms *d* Fixed-end | 14.515 | 0.018 | 5 | **<0.0001** |  |  |  |  |  |  |
|  |  | *T*_0_ *d* Fixed-end | 14.552 | 0.003 | 5 |  |  |  |  |  |  |  |
|  |  | Rest *d*_1,0_ Low-load | 34.13 | 0.75 | 5 | - | nm | Rest vs 6.5ms, 11.5ms, 16.5ms and *T*_0_ for Low-load *d*_1,0_ | Student’s t-test, paired | N/A | Table 2 | N/A |
|  |  | 6.5ms *d*_1,0_ Low-load | 35.16 | 0.71 | 5 | **<0.0001** |  |  |  |  |  |  |
|  |  | 11.5ms *d*_1,0_ Low-load | 35.96 | 0.73 | 5 | **0.0001** |  |  |  |  |  |  |
|  |  | 16.5ms *d*_1,0_ Low-load | 36.10 | 0.65 | 5 | **0.0020** |  |  |  |  |  |  |
|  |  | *T*_0_ *d*_1,0_ Low-load | 36.05 | 0.59 | 5 | **0.0058** |  |  |  |  |  |  |
|  |  | Rest *I*_1,0_ Low-load | 1 | 0 | 5 | - | N/A | Rest vs 6.5ms, 11.5ms, 16.5ms and *T*_0_ for Low-load *I*_1,0_ | Student’s t-test, paired | N/A | Table 2 | N/A |
|  |  | 6.5ms *I*_1,0_ Low-load | 0.96 | 0.03 | 5 | **0.0347** |  |  |  |  |  |  |
|  |  | 11.5ms *I*_1,0_ Low-load | 0.84 | 0.09 | 5 | **0.0169** |  |  |  |  |  |  |
|  |  | 16.5ms *I*_1,0_ Low-load | 0.62 | 0.14 | 5 | **0.0038** |  |  |  |  |  |  |
|  |  | *T*_0_ *I*_1,0_ Low-load | 0.36 | 0.16 | 5 | **0.0009** |  |  |  |  |  |  |
|  |  | Rest *I*_1,1_/*I*_1,0_ Low-load | 0.29 | 0.09 | 5 | - | N/A | Rest vs 6.5ms, 11.5ms, 16.5ms and *T*_0_ for Low-load *I*_1,1_/*I*_1,0_ | Student’s t-test, paired | N/A | Table 2 | N/A |
|  |  | 6.5ms *I*_1,1_/*I*_1,0_ Low-load | 0.44 | 0.11 | 5 | **0.0003** |  |  |  |  |  |  |
|  |  | 11.5ms *I*_1,1_/*I*_1,0_ Low-load | 0.63 | 0.13 | 5 | **0.0003** |  |  |  |  |  |  |
|  |  | 16.5ms *I*_1,1_/*I*_1,0_ Low-load | 0.98 | 0.13 | 5 | **0.0006** |  |  |  |  |  |  |
|  |  | *T*_0_ *I*_1,1_/*I*_1,0_ Low-load | 1.92 | 0.34 | 5 | **0.0005** |  |  |  |  |  |  |
|  |  | Rest *I*_ML1_ Low-load | 1 | 0 | 5 | - | N/A | Rest vs 6.5ms, 11.5ms, 16.5ms and *T*_0_ for Low-load *I*_ML1_ | Student’s t-test, paired | N/A | Table 2 | N/A |
|  |  | 6.5ms *I*_ML1_ Low-load | 0.91 | 0.12 | 5 | 0.163 |  |  |  |  |  |  |
|  |  | 11.5ms *I*_ML1_ Low-load | 0.66 | 0.11 | 5 | **0.0020** |  |  |  |  |  |  |
|  |  | 16.5ms *I*_ML1_ Low-load | 0.27 | 0.10 | 5 | **<0.0001** |  |  |  |  |  |  |
|  |  | *T*_0_ *I*_ML1_ Low-load | 0.01 | 0.08 | 5 | **<0.0001** |  |  |  |  |  |  |
|  |  | Rest *A*_ML1_ Low-load | 1 | 0 | 5 | - | N/A | Rest vs 6.5ms, 11.5ms, 16.5ms and *T*_0_ for Low-load *A*_ML1_ | Student’s t-test, paired | N/A | Table 2 | *A*_ML1_ at *T*_0_ not possible to calculate for one muscle |
|  |  | 6.5ms *A*_ML1_ Low-load | 0.95 | 0.07 | 5 | 0.172 |  |  |  |  |  |  |
|  |  | 11.5ms *A*_ML1_ Low-load | 0.81 | 0.07 | 5 | **0.0027** |  |  |  |  |  |  |
|  |  | 16.5ms *A*_ML1_ Low-load | 0.51 | 0.10 | 5 | **0.0004** |  |  |  |  |  |  |
|  |  | *T*_0_ *A*_ML1_ Low-load | 0.20 | 0.07 | 4 | **0.0004** |  |  |  |  |  |  |
|  |  | Rest *I*_AL1_ Low-load | 1 | 0 | 5 | - | N/A | Rest vs 6.5ms, 11.5ms, 16.5ms and *T*_0_ for Low-load *I*_AL1_ | Student’s t-test, paired | N/A | Table 2 | N/A |
|  |  | 6.5ms *I*_AL1_ Low-load | 0.85 | 0.66 | 5 | 0.631 |  |  |  |  |  |  |
|  |  | 11.5ms *I*_AL1_ Low-load | 0.99 | 0.46 | 5 | 0.946 |  |  |  |  |  |  |
|  |  | 16.5ms *I*_AL1_ Low-load | 1.05 | 0.68 | 5 | 0.879 |  |  |  |  |  |  |
|  |  | *T*_0_ *I*_AL1_ Low-load | 2.58 | 1.64 | 5 | 0.097 |  |  |  |  |  |  |
|  |  | Rest *A*_AL1_ Low-load | 1 | 0 | 5 | - | N/A | Rest vs 6.5ms, 11.5ms, 16.5ms and *T*_0_ for Low-load *A*_AL1_ | Student’s t-test, paired | N/A | Table 2 | N/A |
|  |  | 6.5ms *A*_AL1_ Low-load | 0.86 | 0.37 | 5 | 0.484 |  |  |  |  |  |  |
|  |  | 11.5ms *A*_AL1_ Low-load | 0.97 | 0.23 | 5 | 0.873 |  |  |  |  |  |  |
|  |  | 16.5ms *A*_AL1_ Low-load | 0.98 | 0.35 | 5 | 0.932 |  |  |  |  |  |  |
|  |  | *T*_0_ *A*_AL1_ Low-load | 1.52 | 0.57 | 5 | 0.105 |  |  |  |  |  |  |
|  |  | Rest *I*_M6_ Low-load | 1 | 0 | 5 | - | N/A | Rest vs 6.5ms, 11.5ms, 16.5ms and *T*_0_ for Low-load *I*_M6_ | Student’s t-test, paired | N/A | Table 2 | N/A |
|  |  | 6.5ms *I*_M6_ Low-load | 1.05 | 0.33 | 5 | 0.761 |  |  |  |  |  |  |
|  |  | 11.5ms *I*_M6_ Low-load | 1.04 | 0.32 | 5 | 0.785 |  |  |  |  |  |  |
|  |  | 16.5ms *I*_M6_ Low-load | 1.06 | 0.84 | 5 | 0.882 |  |  |  |  |  |  |
|  |  | *T*_0_ *I*_M6_ Low-load | 1.53 | 1.98 | 5 | 0.686 |  |  | Wilcoxon Signed Rank test |  |  | Shapiro-Wilk P=0.0037 |
|  |  | Rest *S*_M6_ Low-load | 7.176 | 0.003 | 5 | - | N/A | Rest vs 6.5ms, 11.5ms, 16.5ms and *T*_0_ for Low-load *S*_M6_ | Student’s t-test, paired | N/A | Table 2 | N/A |
|  |  | 6.5ms *S*_M6_ Low-load | 7.188 | 0.004 | 5 | **0.0072** |  |  |  |  |  |  |
|  |  | 11.5ms *S*_M6_ Low-load | 7.202 | 0.005 | 5 | **0.0002** |  |  |  |  |  |  |
|  |  | 16.5ms *S*_M6_ Low-load | 7.244 | 0.009 | 5 | **0.0002** |  |  |  |  |  |  |
|  |  | *T*_0_ *S*_M6_ Low-load | 7.280 | 0.006 | 5 | **<0.0001** |  |  |  |  |  |  |
|  |  | Rest *I*_M3_ Low-load | 1 | 0 | 5 | - | N/A | Rest vs 6.5ms, 11.5ms, 16.5ms and *T*_0_ for Low-load *I*_M3_ | Student’s t-test, paired | N/A | Table 2 | N/A |
|  |  | 6.5ms *I*_M3_ Low-load | 0.80 | 0.12 | 5 | **0.02** |  |  |  |  |  |  |
|  |  | 11.5ms *I*_M3_ Low-load | 0.73 | 0.15 | 5 | **0.0175** |  |  |  |  |  |  |
|  |  | 16.5ms *I*_M3_ Low-load | 1.08 | 0.75 | 5 | 0.819 |  |  |  |  |  |  |
|  |  | *T*_0_ *I*_M3_ Low-load | 3.86 | 2.67 | 5 | 0.08 |  |  |  |  |  |  |
|  |  | Rest *A*_M3_ Low-load | 1 | 0 | 5 | - | N/A | Rest vs 6.5ms, 11.5ms, 16.5ms and *T*_0_ for Low-load *A*_M3_ | Student’s t-test, paired | N/A | Table 2 | N/A |
|  |  | 6.5ms *A*_M3_ Low-load | 0.89 | 0.06 | 5 | **0.0213** |  |  |  |  |  |  |
|  |  | 11.5ms *A*_M3_ Low-load | 0.85 | 0.09 | 5 | **0.0226** |  |  |  |  |  |  |
|  |  | 16.5ms *A*_M3_ Low-load | 0.99 | 0.36 | 5 | 0.948 |  |  |  |  |  |  |
|  |  | *T*_0_ *A*_M3_ Low-load | 1.86 | 0.70 | 5 | **0.051** |  |  |  |  |  |  |
|  |  | Rest *L*_M3_ Low-load | 0.06 | 0.02 | 5 | - | N/A | Rest vs 6.5ms, 11.5ms, 16.5ms and *T*_0_ for Low-load *L*_M3_ | Student’s t-test, paired | N/A | Table 2 | N/A |
|  |  | 6.5ms *L*_M3_ Low-load | 0 | 0 | 5 | **0.0024** |  |  |  |  |  |  |
|  |  | 11.5ms *L*_M3_ Low-load | 0.03 | 0.03 | 5 | 0.270 |  |  |  |  |  |  |
|  |  | 16.5ms *L*_M3_ Low-load | 0.26 | 0.05 | 5 | **0.0005** |  |  |  |  |  |  |
|  |  | *T*_0_ *L*_M3_ Low-load | 0.56 | 0.02 | 5 | **<0.0001** |  |  |  |  |  |  |
|  |  | Rest *M*_M3_ Low-load | 0.81 | 0.04 | 5 | - | N/A | Rest vs 6.5ms, 11.5ms, 16.5ms and *T*_0_ for Low-load *M*_M3_ | Student’s t-test, paired | N/A | Table 2 | N/A |
|  |  | 6.5ms *M*_M3_ Low-load | 0.84 | 0.02 | 5 | 0.199 |  |  |  |  |  |  |
|  |  | 11.5ms *M*_M3_ Low-load | 0.82 | 0.02 | 5 | 0.706 |  |  |  |  |  |  |
|  |  | 16.5ms *M*_M3_ Low-load | 0.64 | 0.07 | 5 | **0.0075** |  |  |  |  |  |  |
|  |  | *T*_0_ *M*_M3_ Low-load | 0.41 | 0.02 | 5 | **<0.0001** |  |  |  |  |  |  |
|  |  | Rest *H*_M3_ Low-load | 0.13 | 0.02 | 5 | - | N/A | Rest vs 6.5ms, 11.5ms, 16.5ms and *T*_0_ for Low-load *H*_M3_ | Student’s t-test, paired | N/A | Table 2 |  |
|  |  | 6.5ms *H*_M3_ Low-load | 0.16 | 0.02 | 5 | 0.092 |  |  |  |  |  |  |
|  |  | 11.5ms *H*_M3_ Low-load | 0.15 | 0.01 | 5 | 0.141 |  |  |  |  |  |  |
|  |  | 16.5ms *H*_M3_ Low-load | 0.10 | 0.04 | 5 | 0.104 |  |  |  |  |  |  |
|  |  | *T*_0_ *H*_M3_ Low-load | 0.04 | 0.01 | 5 | **0.0431** |  |  | Wilcoxon signed rank |  |  | Shapiro-Wilk P=0.0434 |
|  |  | Rest *S*_M3_ Low-load | 14.341 | 0.010 | 5 | - | N/A | Rest vs 6.5ms, 11.5ms, 16.5ms and *T*_0_ for Low-load *S*_M3_ | Student’s t-test, paired | N/A | Table 2 | N/A |
|  |  | 6.5ms *S*_M3_ Low-load | 14.312 | 0.007 | 5 | **<0.0001** |  |  |  |  |  |  |
|  |  | 11.5ms *S*_M3_ Low-load | 14.316 | 0.010 | 5 | **0.0001** |  |  |  |  |  |  |
|  |  | 16.5ms *S*_M3_ Low-load | 14.394 | 0.014 | 5 | **0.0007** |  |  |  |  |  |  |
|  |  | *T*_0_ *S*_M3_ Low-load | 14.534 | 0.011 | 5 | **<0.0001** |  |  |  |  |  |  |
|  |  | Rest *d* Low-load | 14.343 | 0.013 | 5 | - | N/A | Rest vs 6.5ms, 11.5ms, 16.5ms and *T*_0_ for Low-load *d* | Student’s t-test, paired | N/A | Table 2 | N/A |
|  |  | 6.5ms *d* Low-load | 14.317 | 0.003 | 5 | **0.0048** |  |  |  |  |  |  |
|  |  | 11.5ms *d* Low-load | 14.322 | 0.006 | 5 | **0.0045** |  |  |  |  |  |  |
|  |  | 16.5ms *d* Low-load | 14.400 | 0.009 | 4 | **0.0009** |  |  |  |  |  |  |
|  |  | *T*_0_ *d* Low-load | 14.550 | 0.007 | 5 |  |  |  |  |  |  |  |
